# Fluorescence-guided laparoscopic repair of ureterosciatic hernia using a self-gripping mesh: a case report

**DOI:** 10.1093/jscr/rjag748

**Published:** 2026-08-31

**Authors:** Junpei Takashima, Hirotoshi Kobayashi, Akitoshi Nankaku, Daisuke Fujimoto, Fumihiko Miura

**Affiliations:** Department of Surgery, Teikyo University School of Medicine, Mizonokuchi Hospital, 5-1-1 Futago, Takatsu-ku, Kawasaki, Kanagawa 213-8507, Japan; Department of Surgery, Teikyo University School of Medicine, Mizonokuchi Hospital, 5-1-1 Futago, Takatsu-ku, Kawasaki, Kanagawa 213-8507, Japan; Department of Surgery, Teikyo University School of Medicine, Mizonokuchi Hospital, 5-1-1 Futago, Takatsu-ku, Kawasaki, Kanagawa 213-8507, Japan; Department of Surgery, Teikyo University School of Medicine, Mizonokuchi Hospital, 5-1-1 Futago, Takatsu-ku, Kawasaki, Kanagawa 213-8507, Japan; Department of Surgery, Teikyo University School of Medicine, Mizonokuchi Hospital, 5-1-1 Futago, Takatsu-ku, Kawasaki, Kanagawa 213-8507, Japan

**Keywords:** ureterosciatic hernia, fluorescent ureteral catheter, self-gripping mesh, laparoscopic repair, hydronephrosis

## Abstract

Ureterosciatic hernia is an exceptionally rare pelvic floor hernia that can cause ureteral obstruction, hydronephrosis, and recurrent urinary tract infection, with no established standard surgical strategy. We report a 75-year-old woman with recurrent pyelonephritis who underwent elective laparoscopic repair after infection control. A near-infrared fluorescent ureteral catheter was placed preoperatively to enable real-time ureteral visualization. Using a transperitoneal three-port approach, the herniated ureter was reduced, and the hernia orifice at the infrapiriform foramen (10 × 15 mm) was reinforced with a self-gripping mesh without additional fixation to minimize neurovascular injury. Fluorescence guidance allowed precise ureteral identification despite pelvic adhesions. Operative time was 90 min with minimal blood loss, and the postoperative course was uneventful, with no recurrence at 1-year follow-up. This fluorescence-guided, non-fixating mesh repair represents a feasible and safety-oriented strategy for this rare condition. To our knowledge, this is the first report of this approach for ureterosciatic hernia.

## Introduction

Sciatic hernia is a rare form of pelvic floor hernia, and ureterosciatic hernia represents an extremely uncommon subtype in which the ureter constitutes the hernia content [1]. Ureteral displacement may result in obstruction, hydronephrosis, recurrent infection, and progressive renal dysfunction [2]. Reported management strategies range from conservative treatment to surgical repair; however, no standardized therapeutic algorithm has been established [1].

Ureteral stenting is widely used to relieve obstruction and may be curative in selected patients. Nevertheless, recurrent infection or persistent symptoms often necessitate definitive surgical repair. Although open surgery was historically the standard approach, minimally invasive techniques, including laparoscopic and robotic repair, have increasingly been reported. The indications for mesh reinforcement and optimal fixation strategies in the deep pelvis remain unclear.

To our knowledge, no previous report has described a fluorescence-guided, non-fixating mesh strategy specifically for ureterosciatic hernia. We report a case of ureterosciatic hernia successfully treated with fluorescence-guided laparoscopic repair using a self-gripping mesh and discuss its potential advantages.

## Case report

**Figure 1:**
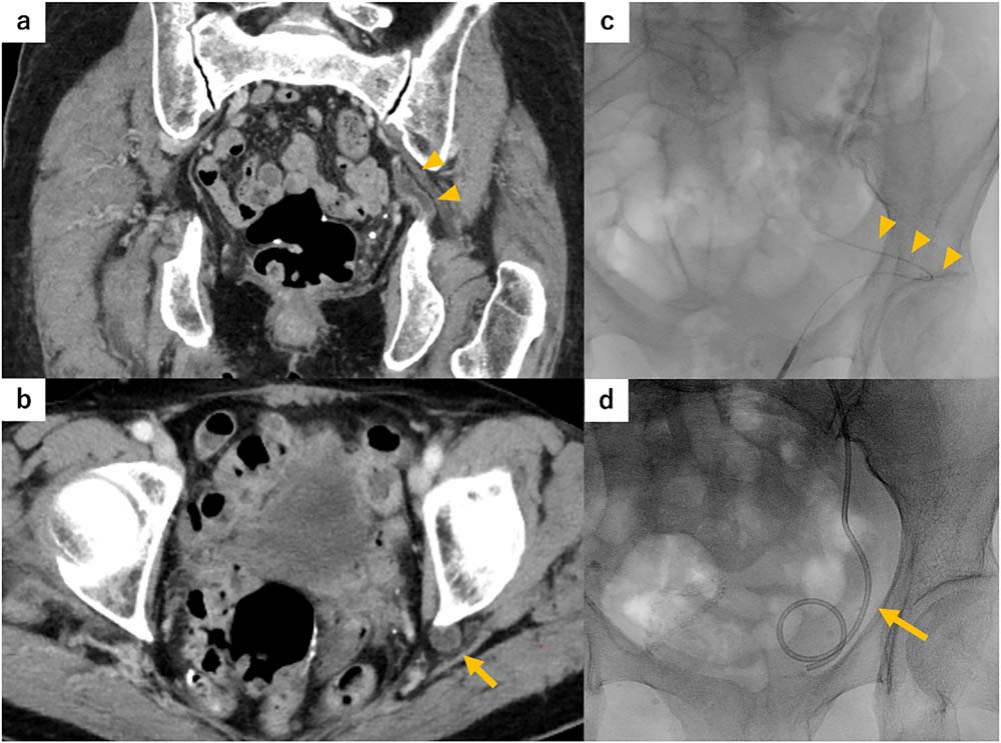
Preoperative imaging and ureteral stenting; (a) contrast-enhanced abdominal CT (coronal view) demonstrating herniation of the left ureter into the sciatic foramen (arrowhead); (b) contrast-enhanced abdominal CT (axial view) showing the left ureter herniating into the sciatic foramen (arrow); (c) fluoroscopic image obtained during insertion of the left ureteral catheter, confirming herniation of the ureter into the sciatic foramen (arrowhead); (d) fluoroscopic image after placement of the left ureteral catheter; the ureteral catheter (arrow) is correctly positioned, with resolution of the herniation.

A 75-year-old woman presented with fever and left flank pain. Her medical history included laparoscopic low anterior resection for rectal cancer 12 years earlier and methotrexate therapy for rheumatoid arthritis. She had a history of recurrent urinary tract infection. On admission, left costovertebral angle tenderness was noted. Urinalysis revealed pyuria and bacteriuria. Laboratory tests demonstrated marked inflammatory response (white blood cell count 22 900/μl; C-reactive protein 29.6 mg/dl). Contrast-enhanced computed tomography (CT) showed left hydronephrosis and suspected herniation of the left ureter through the sciatic foramen (Fig. 1a and b). She was diagnosed with acute pyelonephritis secondary to ureteral obstruction caused by ureterosciatic hernia. A left ureteral stent was placed under fluoroscopic guidance (Fig. 1c). She improved rapidly with antibiotic therapy and was discharged after treatment.

Approximately 1 month after stent removal, she developed recurrent fever and flank pain. Imaging again demonstrated left ureterosciatic hernia with hydronephrosis. Following re-stenting and infection control, elective definitive surgical repair was planned due to recurrent infection attributed to persistent herniation.

### Intraoperative findings

**Figure 2:**
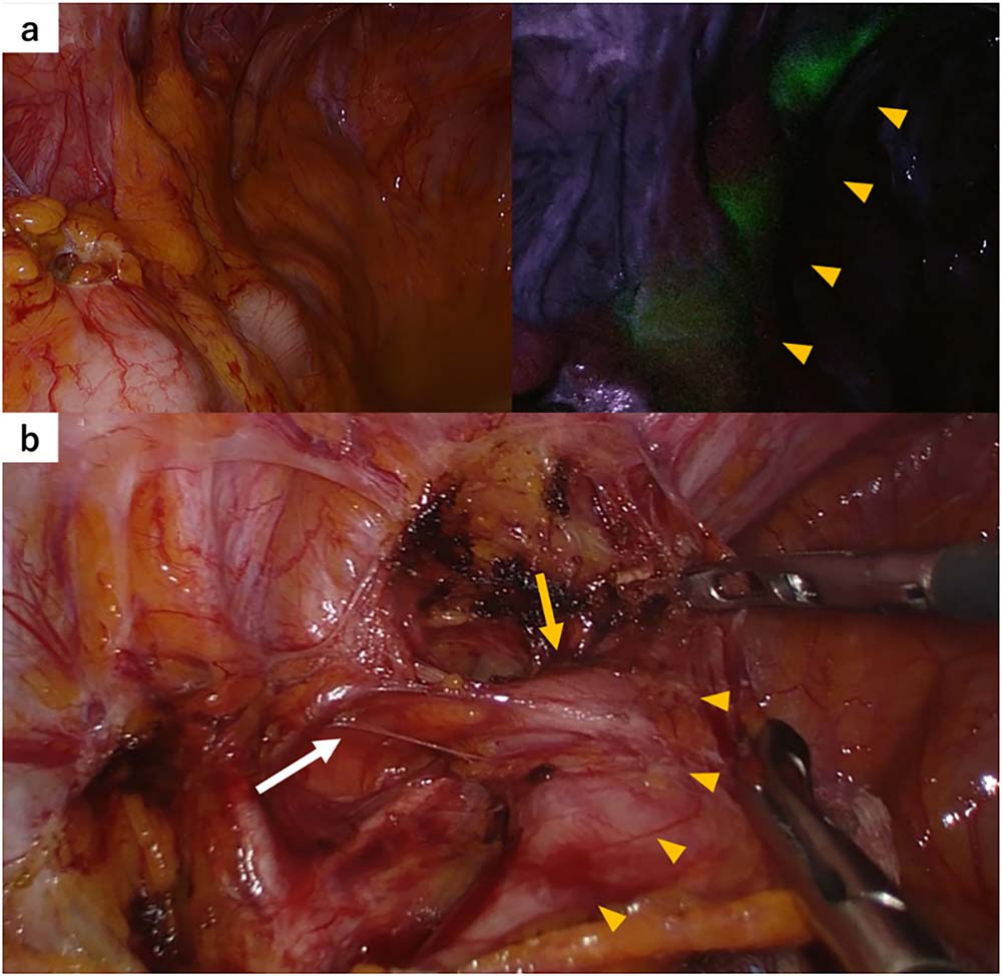
Intraoperative findings and fluorescence-guided ureteral identification; (a) intraoperative view showing adhesion of the rectum to the left pelvic wall; the course of the left ureter was clearly identified using near-infrared fluorescence imaging with a fluorescent ureteral catheter (arrowhead); (b) peritoneal incision and dissection were performed along the course of the ureter (arrowhead); a mild kinking of the ureter was observed (arrow), and a depression corresponding to the greater sciatic foramen was identified adjacent to it (white arrow).

**Figure 3:**
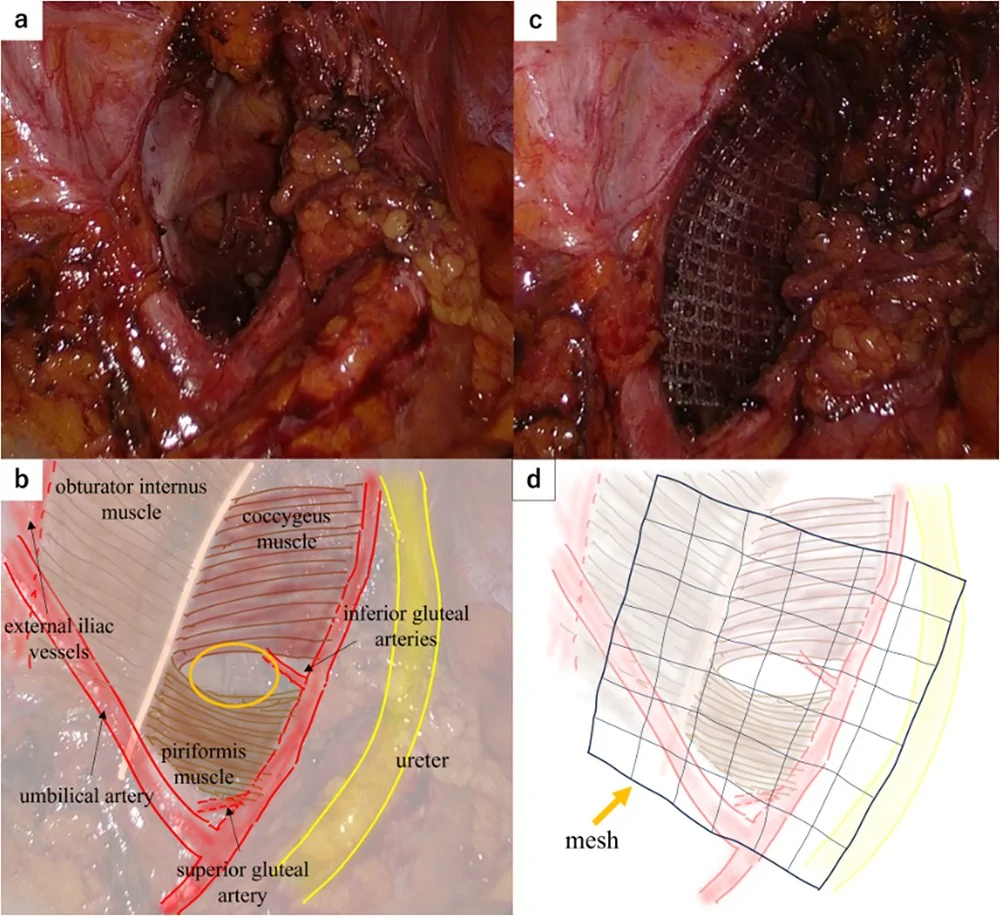
Intraoperative findings and mesh placement; (a) intraoperative view after exposure of the hernia orifice; the defect was located in the infrapiriform foramen (circle), adjacent to the coccygeus muscle, piriformis muscle, and inferior gluteal vessels; (b) schematic illustration corresponding to panel (a), demonstrating the anatomical relationships around the hernia orifice; (c) intraoperative view after placement of a 50 × 50 mm self-gripping mesh centered over the hernia defect; no additional fixation was required; (d) schematic representation of mesh positioning (arrow), illustrating coverage of the hernia orifice and surrounding anatomical landmarks.

To facilitate ureteral identification, a near-infrared fluorescent ureteral catheter (NIRC®, Cardinal Health, Tokyo, Japan) was placed preoperatively. Surgery was performed under general anesthesia using a transperitoneal laparoscopic approach. Three ports were utilized: a 12-mm camera port at the umbilicus, a 12-mm working port in the right lower abdomen, and a 5-mm assistant port in the right flank. Before peritoneal incision, near-infrared fluorescence mode was used to visualize the ureter into the deep pelvis. During the procedure, mild pelvic adhesions—likely related to prior rectal surgery—were observed; however, the left ureter was clearly visualized under fluorescence, allowing for safe adhesiolysis (Fig. 2a). The lateral peritoneum was incised along the ureter, and dissection was advanced toward the greater sciatic foramen. Throughout this process, particular attention was paid to the surrounding neurovascular structures. The ureter demonstrated mild angulation as it descended toward the pelvic floor, and a depression corresponding to the greater sciatic foramen was identified posterolateral to the ureter (Fig. 2b). The hernia orifice was confirmed to pass through the infrapiriformis compartment, measuring ~10 × 15 mm (Fig. 3a). After reduction of the hernia contents and sufficient circumferential dissection, a 50 × 50 mm self-gripping mesh (ProGrip™, Medtronic, Minneapolis, MN, USA) was positioned to cover the defect (Fig. 3b). Given the risk of neurovascular injury, we intentionally avoided suture or tack fixation; reinforcement was achieved solely through the mesh’s self-gripping mechanism. Finally, the peritoneum was closed with a continuous 3–0 barbed suture (V-Loc™, Medtronic). The total operative time was 90 min with minimal blood loss.

### Postoperative course

**Figure 4:**
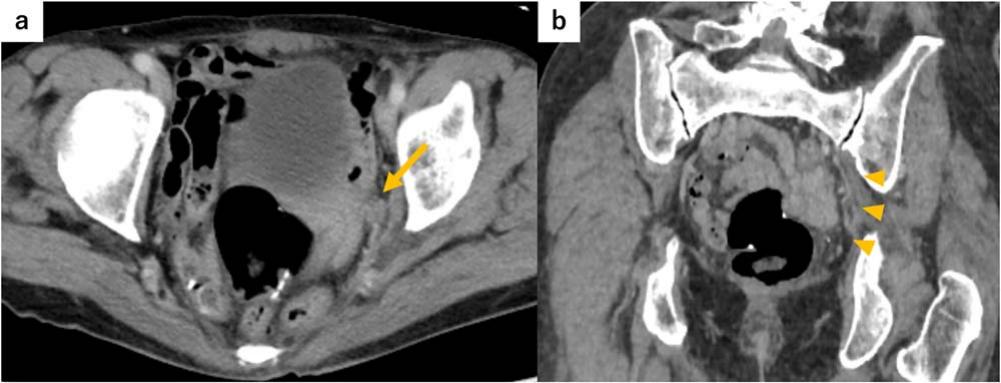
One-year postoperative CT findings without evidence of recurrent ureterosciatic hernia; (a) axial contrast-enhanced CT image obtained 1 year postoperatively showing no evidence of recurrent herniation of the ureter into the sciatic foramen (arrow); (b) coronal contrast-enhanced CT image obtained 1 year postoperatively demonstrating complete resolution of the sciatic hernia (arrowhead).

The postoperative course was uneventful. The patient was discharged on postoperative Day 4. At the 1-year follow-up, she remained asymptomatic without evidence of recurrence (Fig. 4a and b).

## Discussion

Ureterosciatic hernia is an exceptionally rare pelvic floor hernia, and standardized management strategies have not been established. In this report, we describe a case successfully treated using fluorescence-guided laparoscopic repair with a self-gripping mesh.

Among the 48 cases reported in the literature, conservative and surgical treatments have been described with similar frequency. However, the optimal surgical technique, indications for mesh use, and fixation methods remain controversial. Of the reported surgically treated cases, mesh reinforcement was performed in only a minority, and detailed descriptions of mesh type and fixation technique are limited (Table 1) [2–11]. Various fixation methods, including sutures, tacks, fibrin glue, or no fixation, have been described; however, no study has systematically evaluated the anatomical risks associated with each fixation technique. To the best of our knowledge, no procedure-specific study has demonstrated that mesh fixation significantly increases the risk of neurovascular injury during ureterosciatic hernia repair. However, the sciatic foramen, particularly the infrapiriform compartment, contains several critical neurovascular structures, including the sciatic nerve, inferior gluteal vessels, inferior gluteal nerve, and internal pudendal vessels and nerve. Therefore, penetrating fixation using sutures or tacks may theoretically increase the risk of iatrogenic neurovascular injury in this anatomically crowded region. Moreover, in laparoscopic surgery, where visualization and working angles are inherently limited, safe penetrating fixation may be technically more challenging. In contrast, evidence from groin hernia surgery has demonstrated that mesh reinforcement reduces recurrence compared with non-mesh repair [12]. Furthermore, self-gripping mesh has been reported to provide recurrence outcomes comparable to those of conventionally fixed mesh while eliminating the need for additional fixation, shortening operative time, and reducing early postoperative pain [13]. Current international guidelines for groin hernia management also recognize self-gripping mesh as an acceptable option in appropriately selected patients [14]. Although direct evidence for ureterosciatic hernia is lacking, these findings from groin hernia surgery provide indirect support for mesh reinforcement without additional penetrating fixation. Based on the anatomical complexity of the sciatic foramen and the available indirect evidence from groin hernia surgery, we selected a self-gripping mesh to preserve the principle of mesh reinforcement while minimizing potential injury to the adjacent neurovascular structures by avoiding penetrating fixation. This strategy may represent a reasonable balance between reinforcement and safety during hernia repair within the anatomically complex deep pelvis.

**Table 1:** Summary of published surgical cases of ureterosciatic hernia with emphasis on mesh use and fixation strategies.

Author	Year	Age	Gender	Side	Hernia location	Surgical approach	Mesh material (product)	Mesh fixation method	Mesh size
Gee *et al*. [3]	1999	60	Female	Left	NR	Laparo	Marlex mesh	Stapler	NR
Touloupidis *et al*. [4]	2006	61	Female	Right	Infra piriformis	Open	NR	NR	NR
Witney-Smith *et al*. [2]	2007	59	Female	Left	NR	Laparo	Prolene mesh + plug	No fixation	NR
Whyburn and Alizadeh [5]	2013	74	Female	Bilateral	NR	Laparo	NR	NR	NR
Wai *et al*. [6]	2016	68	Female	Left	NR	Open	NR	NR	NR
Rose *et al*. [7]	2020	68	Female	Left	Supra piriformis	Robot	Bioavailable mesh	Fibrin sealant fixation	4 × 4 cm
Kubota *et al*. [8]	2020	85	Female	Left	NR	Laparo	NR	NR	NR
Li *et al*. [9]	2022	72	Female	Right	NR	Laparo	SIS® patch (biological mesh)	No fixation	NR
Fridling *et al*. [10]	2023	73	Female	Left	Supra piriformis	Robot	Bio-A® (biosynthetic absorbable mesh)	Fibrin sealant fixation	4 × 4 cm
García-Rico *et al*. [11]	2024	62	Female	Right	NR	Laparo	NR	NR	NR
Present case	2026	75	Female	Left	Infra piriformis	Laparo	ProGrip™ (self-gripping mesh)	No fixation	5 × 5 cm

NR, not reported; Laparo, laparoscopic; Robot, robotic.

Another key aspect of our strategy was fluorescence-guided ureteral identification. Several previous reports have described ureterosciatic hernia repair with preoperative ureteral stenting, and successful surgical repair using a conventional ureteral stent has been reported. However, to the best of our knowledge, the use of near-infrared fluorescence guidance during ureterosciatic hernia repair has not previously been described. Furthermore, we found no published reports of intraoperative ureteral injury during ureterosciatic hernia repair performed with conventional ureteral stents. Therefore, based on the currently available evidence, it cannot be concluded that fluorescent ureteral catheters are superior to conventional ureteral stents. To the best of our knowledge, only two previously reported patients had a history of prior pelvic surgery, both of whom had undergone gynecologic procedures [6, 15]. No cases have been reported following rectal surgery, and none of these reports described intraoperative pelvic adhesions. In contrast, our patient had previously undergone laparoscopic low anterior resection for rectal cancer, and dense postoperative pelvic adhesions were anticipated. Intraoperatively, dense adhesions involving the left pelvic wall were encountered. Under fluorescence guidance, the ureter was continuously visualized from the early stage of the operation, allowing safe adhesiolysis while maintaining clear visualization of the ureteral course. This facilitated safe dissection within the anatomically altered pelvis. However, fluorescent ureteral catheters are associated with additional costs compared with conventional ureteral stents. Therefore, we do not advocate their routine use in all cases of ureterosciatic hernia. Rather, this technique may be particularly beneficial in selected patients in whom ureteral identification is expected to be technically challenging, such as those with previous pelvic surgery, recurrent inflammation secondary to ureterosciatic hernia, or severe pelvic adhesions following radiotherapy. Continuous intraoperative visualization of the ureter may enhance surgical safety in these situations. Given the increasing adoption of fluorescence imaging in minimally invasive surgery, its application may represent a useful option for selected patients with rare deep pelvic hernias. However, its cost-effectiveness remains to be established and warrants further investigation.

This report has several limitations. First, it describes a single case, and long-term recurrence data are lacking. Second, the appropriate mesh size and the indications for mesh reinforcement remain undefined because of the extremely limited number of reported cases. Furthermore, the size of the hernia defect has not been described in previous reports [2–11]. In the present case, the hernia defect measured ~10 × 15 mm, which may provide useful anatomical information for future surgical planning. Additional case accumulation is needed to establish appropriate indications for mesh reinforcement and mesh sizing. Nevertheless, our experience suggests that fluorescence-guided ureteral identification combined with self-gripping mesh repair may represent a rational surgical strategy for ureterosciatic hernia.

## Conclusion

Fluorescence-guided, non-fixating mesh repair may represent a safe and rational minimally invasive strategy for ureterosciatic hernia. In the anatomically constrained environment of the deep pelvis, combining enhanced ureteral visualization with fixation-free reinforcement may help optimize surgical safety.

## Data Availability

Data sharing is not applicable to this article as no data sets were generated or analyzed during the current study.
